# Supplementary material for: Sorting at embryonic boundaries requires high heterotypic interfacial tension
Source: Nat Commun. 2017 Jul 31;8:157. doi: 10.1038/s41467-017-00146-x (PMC5537356; doi:10.1038/s41467-017-00146-x)
Supplement: Supplementary file 2 — Supplementary Software 1 [file 41467_2017_146_MOESM2_ESM.zip › PottsModel/SrcPottsModel/doc/model/VectorUtils.html]

VectorUtils


JavaScript is disabled on your browser.


Skip navigation links


- Overview
- Package
- Class
- Use
- Tree
- Deprecated
- Index
- Help

- Prev Class
- Next Class

- Frames
- No Frames

- All Classes

- Summary:
- Nested |
- Field |
- Constr |
- Method

- Detail:
- Field |
- Constr |
- Method


model

## Class VectorUtils

- java.lang.Object
- - model.VectorUtils

- ---

    

  ```
  public class VectorUtils
  extends java.lang.Object
  ```

- - ### Constructor Summary

    Constructors

    Constructor and Description

    `VectorUtils()`
  - ### Method Summary

    All Methods Static Methods Concrete Methods

    | Modifier and Type | Method and Description |
    | --- | --- |
    | `static double` | `getAngleFromHorizontal(java.awt.Point pivot, java.awt.Point point)` Gives angle from horizontal of a point with respect to the |
    | `static double` | `getAngleFromVertex(java.awt.Point p1, java.awt.Point p2, java.awt.Point p3)` |
    | `static double` | `measureAngle(Vector pVector, java.awt.Point pPoint)` |

    - ### Methods inherited from class java.lang.Object

      `equals, getClass, hashCode, notify, notifyAll, toString, wait, wait, wait`

- - ### Constructor Detail


    - #### VectorUtils

      ```
      public VectorUtils()
      ```
  - ### Method Detail


    - #### getAngleFromHorizontal

      ```
      public static double getAngleFromHorizontal(java.awt.Point pivot,
                                                  java.awt.Point point)
      ```

      Gives angle from horizontal of a point with respect to the

      Parameters:
      :   `pivot` -
      :   `point` -

      Returns:


    - #### measureAngle

      ```
      public static double measureAngle(Vector pVector,
                                        java.awt.Point pPoint)
      ```

      Parameters:
      :   `pHalfLine` -
      :   `pPoint` -

      Returns:
      :   angle (from 0 to PI), always positive


    - #### getAngleFromVertex

      ```
      public static double getAngleFromVertex(java.awt.Point p1,
                                              java.awt.Point p2,
                                              java.awt.Point p3)
      ```


Skip navigation links


- Overview
- Package
- Class
- Use
- Tree
- Deprecated
- Index
- Help

- Prev Class
- Next Class

- Frames
- No Frames

- All Classes

- Summary:
- Nested |
- Field |
- Constr |
- Method

- Detail:
- Field |
- Constr |
- Method
